# Surgical outcomes of proximal femoral bone cysts in pediatric patients: a retrospective study of 41 cases

**DOI:** 10.3389/fped.2024.1331089

**Published:** 2024-06-24

**Authors:** Taichun Li, Zhenzhen Dai, Qichao Ma, Han Zhou, Hai Li, Ziming Zhang

**Affiliations:** ^1^ Shanghai Children's Hospital, School of Medicine, Shanghai Jiao Tong University, Shanghai, China; ^2^ Xinhua Hospital, School of Medicine, Shanghai Jiao Tong University, Shanghai, China

**Keywords:** children, pathological fracture, surgical intervention, bone cyst, proximal femur

## Abstract

**Purpose:**

The aim of this study was to evaluate the surgical outcomes of proximal femoral bone cysts in pediatric patients.

**Methods:**

We retrospectively analyzed 41 pediatric patients (31 males and 10 females, mean age 7.47 ± 2.67 years, range 2.03–14.67 years) diagnosed with proximal femoral bone cysts treated at a single institute between March 2009 and November 2021. Data included demographics, preoperative details, intraoperative conditions, surgical techniques, postoperative outcomes, recurrence, and complications.

**Results:**

Of the participants, 68% presented with simple bone cysts and 32% with aneurysmal bone cysts. Prior to surgery, 32% exhibited pathological fractures. Surgical methods included lesion curettage, defect filling using allograft bone and Minimally-Invasive Injectable Graft ×3, and varied fixation techniques. Postoperative recurrence (17%) was associated with cyst location between the capital femoral epiphysis and the linea intertrochanterica (*P* = 0.010). At the final assessment (mean follow-up: 26.51 ± 18.99 months), all showed radiological bony union with 93% rated as “good” and 7% as ‘fair’ based on Ratliff hip scores. Complications arose in 20% of patients, significantly correlated with prior pathological fractures (*P* = 0.007) and their association with the linea intertrochanterica (*P* = 0.004). Those with fractures reported higher intraoperative blood loss (*P* = 0.015) and longer surgery durations (*P* = 0.012) compared to those without.

**Conclusion:**

Treating pediatric proximal femoral bone cysts using techniques such as lesion curettage, defect filling, and selective internal fixation yields favorable outcomes. The presence of pathological fractures can prolong surgical time, increase intraoperative blood loss, and elevate postoperative complication risks. Hence, early surgical intervention for these cysts is recommended to prevent fractures.

## Introduction

Bone cysts, characterized as benign osteolytic lesions, are most commonly observed in children and adolescents. The primary sites of manifestation are the proximal humerus and femur (1). Treatment objectives for these cysts include preventing pathologic fractures, promoting healing, and minimizing recurrence (2). Currently, strategies to promote cyst healing encompass a range of interventions, including steroid injections, curettage and grafting, internal fixation, or a combination thereof. Measures aimed at mitigating recurrence risk and constraining the scope of recurrence entail employing diverse approaches, such as high-speed burr, phenol application, sclerotherapy, cryotherapy, argon beam coagulation, and the utilization of synthetic bone substitutes, among others. Furthermore, pharmacological agents like polidocanol are frequently employed in embolization therapy for aneurysmal bone cyst (ABC) (1). Notably, bone cysts are the leading cause of pathologic fractures in pediatric patients, with the proximal femur being the secondary most common site of fracture (3, 4).

During weight-bearing activities, the pertrochanteric and subtrochanteric regions of the proximal femur experience significant tension and bending forces. Consequently, fractures in these regions can lead to complications such as coxa vara, limb length discrepancies, and avascular necrosis (AVN) of the femoral head (5). These complications underscore the challenge pediatric surgeons face when managing proximal femoral bone cysts (6).

There's a continuing debate over the optimal treatment strategy for proximal femoral bone cysts in the pediatric population (5–10). The use of Flexible Intramedullary Nails (FINs) has been proposed to disrupt the cyst wall, thus promoting healing by allowing cyst fluid mobility and reducing wall pressure (11). Similarly, the Locking Compression Pediatric Hip Plate (LCP-PHP) was designed to improve the safety and efficacy of both inter- and subtrochanteric osteotomies, as well as to aid in the management of femoral neck fractures (12). However, literature addressing the efficacy of ESINs or LCP-PHP in the internal fixation of these cysts remains scarce. With our institution's retrospective data, we aim to provide valuable insights into the treatment outcomes and methodologies.

## Materials and methods

With the endorsement of our institution's review board, we conducted a retrospective review of patient records spanning from 2009 to 2021. Criteria for inclusion comprised of male patients below 16 years and female patients under 14 years, with a definitive diagnosis of proximal femoral bone cyst and pathological confirmation of either simple bone cyst (SBC) or ABC. Exclusions encompassed patients with incomplete records, a postoperative follow-up of less than 12 months, or a closed epiphyseal plate at intervention time.

## Data collection

We procured data from the institutional databases, extracting demographics, clinical specifics, and imaging details. Metrics encompassed patient age, gender, cyst details (side, size, location), pathology category, fixation method, operative details (time, blood loss, transfusion), and outcomes(healing, treatment duration, follow-up, recurrence, complications). Treatment duration was defined from implant to internal fixation removal, excluding biopsy and bone graft instances. Active bone cysts were defined as those <1 cm from the epiphysis edge (1). Bone cysts in the proximal femur, with a bone cyst index (cyst area/square diameter of diaphysis) >3.5 (13), or occupying >85% of a long bone's diameter (14), were labeled as high fracture risk. Treatment strategies followed Erol et al.'s classification (7).

## Surgical procedures

• Biopsy + Curettage + Bone Grafting: Employing a modified Watson-Jones approach (15), a longitudinal incision from the greater trochanter is created in line with the femur's orientation. After establishing a cyst or fracture window and removing the lesion, the void is filled with allograft bone and Minimally-Invasive Injectable Graft X3 (MIIG X3, Wright Medical Technology).
• Biopsy + Curettage + FIN + Bone Grafting: After the initial approach, FIN placement adheres to Metaizeau's technique (16), with two 2.5–3.5 mm FINs (AO Synthes®) retrogradely inserted under C-arm fluoroscopy. Post-FIN insertion, the site is packed with allograft bone and MIIG X3.
• Biopsy + Curettage + LCP-PHP (+ K-Wires) + Bone Grafting: Using the modified Watson-Jones strategy, LCP-PHP guidelines (12) informed the fixation. K-wires added stability for particular cysts with an epiphyseal edge distance under 1 cm. Direct observation ensured accurate fracture realignment.

## Postoperative management and follow-up

After surgery, the impacted limb is secured using either a hip spica cast or brace for a period of six weeks. Subsequent to its removal, patients begin a phased regimen of weight-bearing exercises. Scheduled follow-ups occur at 6 weeks, 3, 6, 9, and 12 months post-operation, and then annually. X-rays are used to monitor the bone cyst healing process and the union of fractures.

The prognosis derives from post-surgical imaging results. These are categorized following the Neer Classification system as modified by Chang et al. (17). This system identifies four healing stages: Healed (Grade I), Healing with a defect (Grade II), Persistent cyst (Grade III), and Recurrent cyst (Grade IV). Both Grades III and IV typically necessitate further treatment. A coxa vara deformity is described as a femoral neck-shaft angle under 120° (18). The Ratliff criteria, assessing pain, mobility, activity, and radiological evidence, determine the treatment's final outcome, which can be categorized as either good (clinically, no or negligible pain, full or minimal restrictive hip movement, and normal activity or the avoidance of games. Normal or some deformity of the femoral neck in the radiograph), fair (clinically, occasional pain, hip movement restriction less than 50%, and normal activity or the avoidance of games. Severe deformity of the femoral neck, mild avascular necrosis in the radiograph), or poor (clinically, disabling pain, hip movement restriction more than 50%, and restricted activity. Severe AVN, degenerative arthritis, arthrodesis in the radiograph) (19).

## Statistical analysis

We utilized IBM SPSS Statistics 22.0 for data assessment. Quantitative datasets underwent evaluation via independent-samples t-test or Mann-Whitney *U* test. Categorical sets were analyzed using the chi-square or Fisher's exact test. A *P*-value below 0.05 was considered significant.

## Results

**Figure 1:**
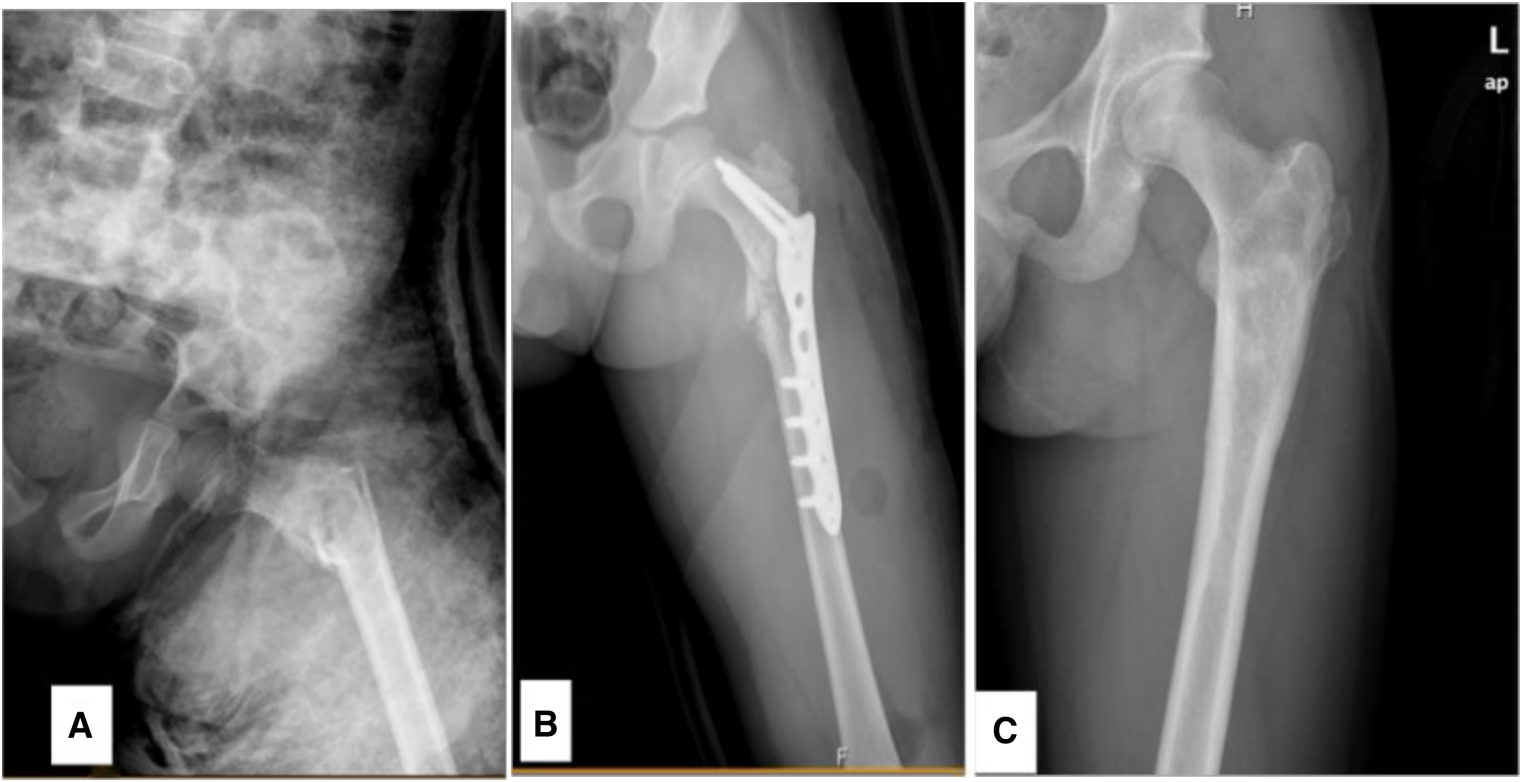
An 8.9-year-old male diagnosed with a SBC in the proximal left femur concomitant with a fracture. (**A**) Preoperative radiograph. (**B**) Radiograph taken 3 days following biopsy, curettage, LCP-PHP procedure, and bone grafting. (**C**) Radiograph captured 6.2 years post-surgery.

**Figure 2:**
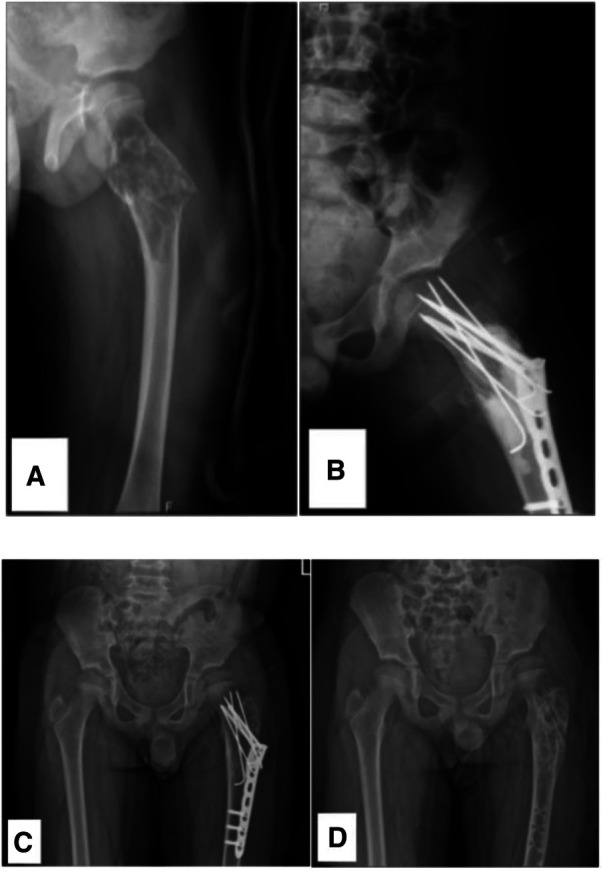
A 7.8-year-old male diagnosed with a SBC in the proximal left femur with an associated fracture. (**A**) Preoperative radiograph. (**B**) Radiograph taken 3 days post-biopsy, curettage, LCP-PHP, K-Wires procedure, and bone grafting. (**C**, **D**) Radiographs at 1.8 years post-surgery and following the removal of the internal fixation. As time progresses and the patient's femoral neck undergoes growth and development, the K-wire initially placed across the epiphyseal plate now appears to be positioned outside it.

**Figure 3:**
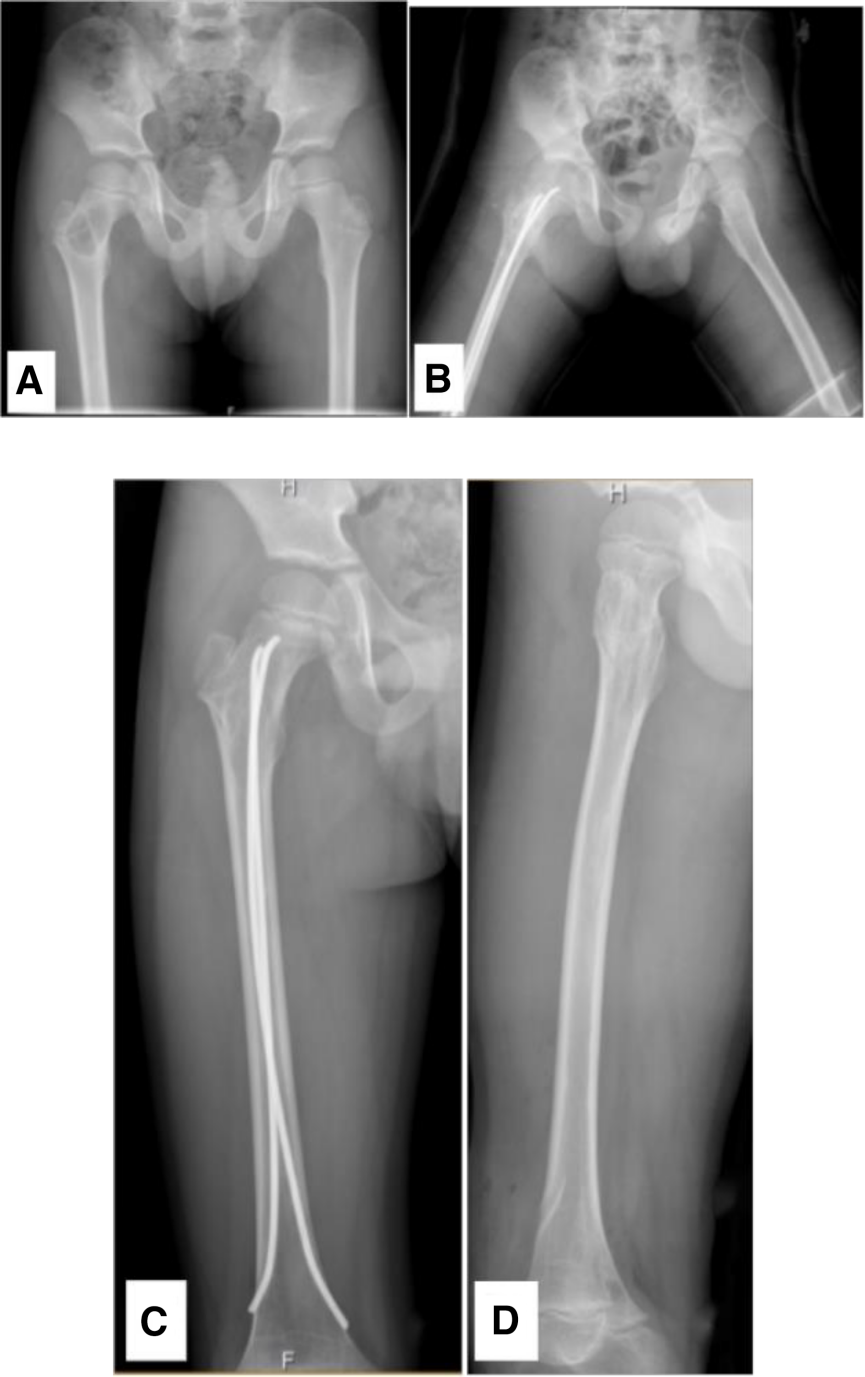
A 6.28-year-old male diagnosed with a SBC in the proximal right femur. (**A**) Preoperative radiograph. (**B**) Radiograph taken 3 days post-biopsy, curettage, FIN procedure, and bone grafting. (**C**, **D**) Radiographs at 1.4 years post-surgery and following the removal of the internal fixation.

In total, 41 patients were incorporated into the final analysis, 31 of whom were male and 10 female. At the time of surgery, the average age was 7.47 ± 2.67 years, ranging from 2.03 to 14.67 years. The average follow-up duration was 26.51 ± 18.99 months, with a range of 12.20 to 82.13 months. Diagnostically, 28 patients (68%) presented with SBCs, while 13 (32%) had ABCs. Thirteen patients (32%) exhibited active bone cysts, and 28 (68%) had latent cysts. Treatment modalities varied: 31 patients were stabilized with LCP-PHP (Figures 1 and 2), 7 with FINs (Figure 3), and 3 underwent only biopsy and bone grafting (Table 1). The incidence of pathological fractures was 32% (13/41), where 12 were stabilized with LCP-PHP and one with FINs. All patients achieved lesion healing (modified Neer grade I or II) post-treatment. However, 7 out of 41 required additional curettage and bone grafting due to persistent or recurrent lesions (modified Neer grade III or IV). According to the Ratliff hip scores, 38 cases (93%) reported good outcomes, while 3 cases (7%) had fair results.

**Table 1:** Demographics and postoperative outcomes of 41 patients.

Parameter	Numeric value
Age at surgery (years)	7.47 ± 2.67
Gender	
Male	31 (76%)
Female	10 (24%)
Affected side	
Left	17 (41%)
Right	24 (59%)
Involvement of linea intertrochanterica	
Yes	21 (51%)
No	20 (49%)
Disease stage	
Active	13 (32%)
Latent	28 (68%)
Pathology type	
SBC	28 (68%)
ABC	13 (32%)
Presence of pathologic fracture	
Yes	13 (32%)
No	28 (68%)
Mode of internal fixation	
None	3 (7%)
FINs	7 (17%)
LCP-PHP	19 (46%)
LCP-PHP & K-wires	12 (29%)
Number of recurrent cases	7 (17%)
Number of patients with complications	8 (20%)
Length of follow-up (months)	26.51 ± 18.99

Values are presented as the mean ± SD (range) or frequency (percentage).

Upon categorizing 41 cases based on the preoperative presence of pathological fractures, it was observed that bone cysts involving the linea intertrochanterica had a higher likelihood of coinciding with pathological fractures (*P* < 0.05). Patients with these fractures (*n* = 13) demonstrated an average surgery duration of 118.23 ± 28.77 min and an intraoperative blood loss of 269.23 ± 266.57 ml. Notably, 69.2% (9/13) of this subset needed intraoperative blood transfusion. In contrast, those without pathological fractures (*n* = 28) averaged a surgery duration of 95.79 ± 23.64 min with a blood loss of 58.21 ± 52.64 ml (*P* < 0.05). A mere 3.6% (1/28) among them required intraoperative blood transfusion (*P* < 0.05) (refer to Table 2).

**Table 2:** Comparative analysis based on the presence or absence of pathological fractures pre-surgery.

Parameter	Pathological fracture (*n* = 13)	No pathological fracture (*n* = 28)	*P* value
Age at surgery (years)	8.13 ± 1.91	7.16 ± 2.94	0.286
Gender [female, no. (%)]	3 (23.1)	7 (25.0)	>0.999
Stage [active, no. (%)]	3 (23.1)	10 (35.7)	0.493
Cyst location [involvement of linea intertrochanterica, no. (%)]	11 (84.6)	10 (35.7)	0.004
Pathology type (ABC, no. [%])	5 (38.5)	8 (28.6)	0.720
Preoperative RBC (10^12^/L)	4.44 ± 0.59	4.61 ± 0.39	0.302
Preoperative HGB (g/L)	123.46 ± 16.19	129.04 ± 9.57	0.174
Preoperative Hct (%)	36.46 ± 4.56	37.86 ± 2.94	0.244
Operative time (min)	118.23 ± 28.77	95.79 ± 23.64	0.012
Intraoperative blood loss (ml)	269.23 ± 266.57	58.21 ± 52.64	0.015
Intraoperative blood transfusion (ml)	165.38 ± 141.99	5.36 ± 28.35	0.002
Number of recurrent cases [no. (%)]	2 (15.4)	5 (17.9)	>0.999
Number of patients with complications [no. (%)]	6 (46.2)	2 (7.1)	0.007
Follow-up duration (months)	31.76 ± 23.36	24.07 ± 16.51	0.232

RBC, red blood cell; HGB, hemoglobin; Hct, hematocrit.

**Figure 4:**
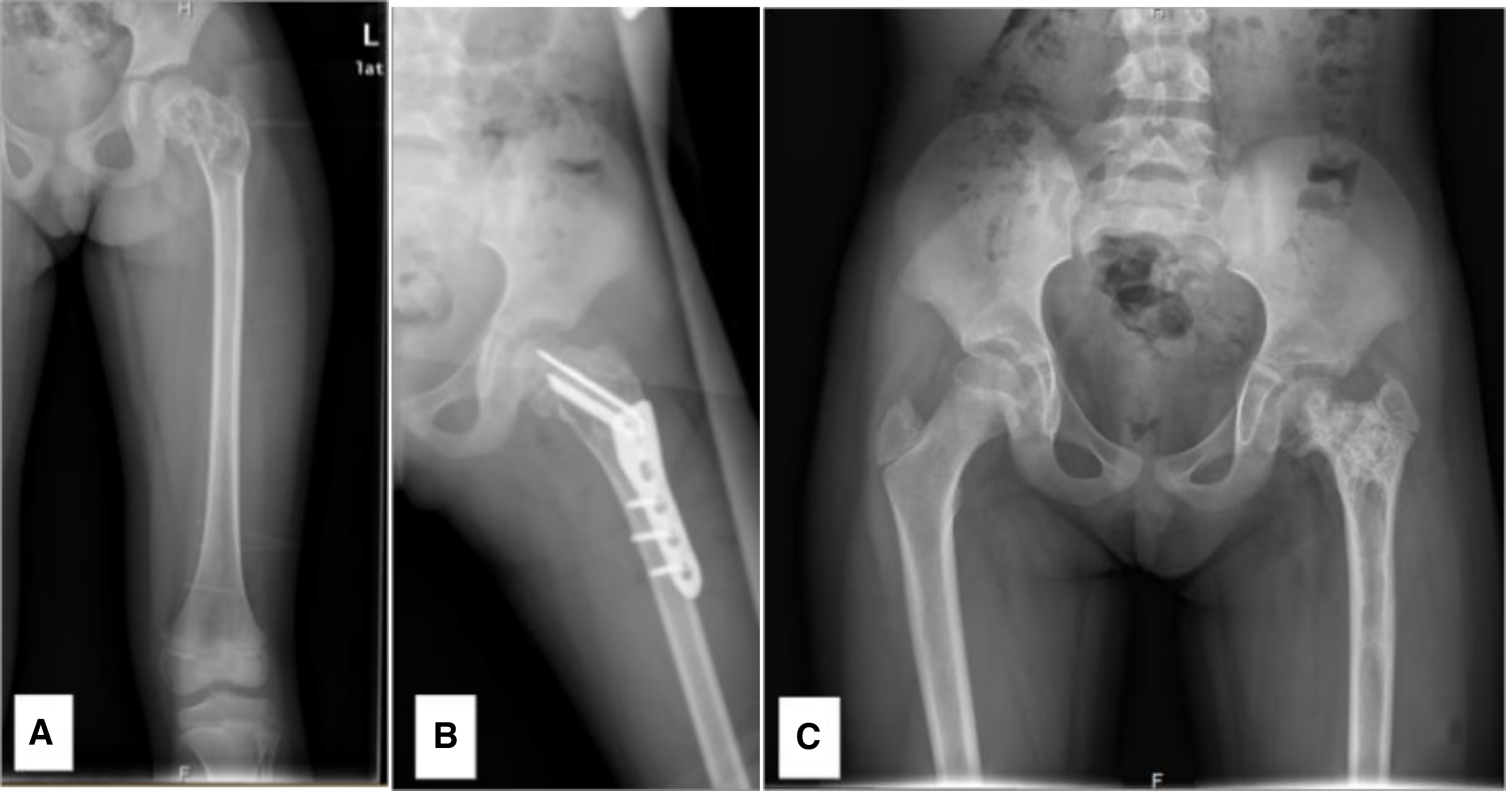
A 6.76-year-old female diagnosed with a SBC in the proximal left femur, combined with a fracture. (**A**) Preoperative radiograph. (**B**) Radiograph taken 3 days post-biopsy, curettage, LCP-PHP procedure, and bone grafting. (**C**) Radiograph at 1.9 years post-surgery, revealing premature closure of the epiphysis, shortening of the femoral neck, and coxa vara.

**Figure 5:**
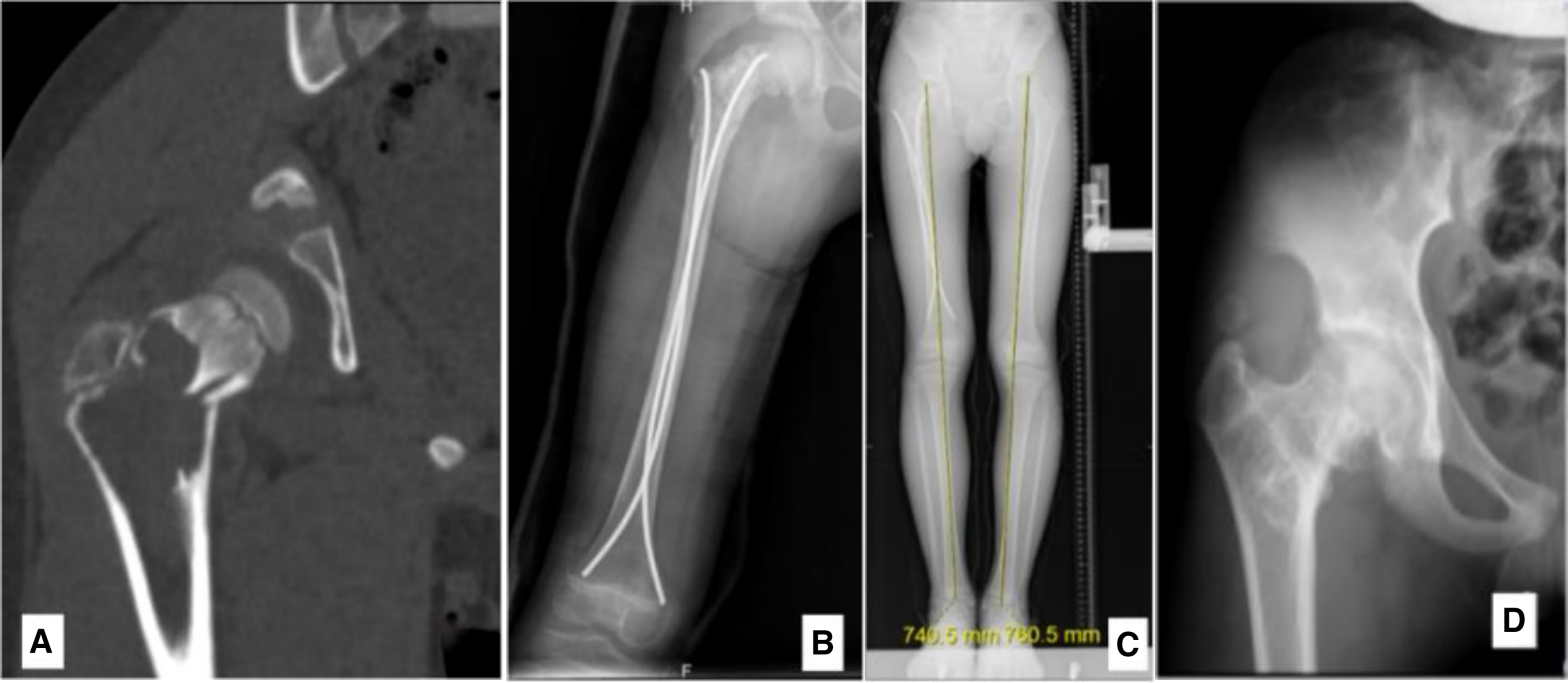
An 8.7-year-old male diagnosed with an ABC in the right proximal femur, accompanied by a fracture. (**A**) Preoperative CT scan, coronal section. (**B**) Radiograph taken 3 days post-biopsy, curettage, FIN procedure, and bone grafting. (**C**) Radiograph at 2.2 years post-surgery showing LLD with the right femur being 2 cm shorter. (**D**) Radiograph at 5.4 years post-surgery, highlighting premature closure of the epiphysis, deformity due to femoral neck shortening, elevated position of the greater trochanter (high riding), and coxa vara.

In this investigation, 14 complications were identified across 8 patients. Specifically, 7 patients presented with a leg length discrepancy (LLD) of 20 mm or less, while 2 exhibited coxa vara and femoral neck shortening deformities. Additionally, premature epiphyseal closure was noted in 2 patients, and a high riding greater trochanter was observed in another (Figures 4, 5). Such manifestations—LLD, coxa vara, a high riding greater trochanter, premature closure of the epiphysis, and femoral neck shortening—were classified as post-surgical morphological disturbances in the proximal femur.

Upon examining determinants of post-surgical morphological disturbances in the proximal femur, our analysis revealed that cases with preoperative pathological fractures exhibited a heightened susceptibility to these post-surgical morphological disturbances in the proximal femur (*P* < 0.05) (Table 3).

**Table 3:** Analysis of risk factors of complication of all 41 patients.

	Non-growth plate disorganization (*n* = 33)	growth plate disorganization (*n* = 8)	*P* value
Age at surgery (years)	7.45 ± 2.76	7.55 ± 2.47	0.923
Gender [female, no. (%)]	6 (18%)	4 (50%)	0.082
Stage [active, no. (%)]	11 (33%)	2 (25%)	>0.999
Pathology type [ABC, no. (%)]	11 (33%)	2 (25%)	>0.999
Cyst location [involvement of linea intertrochanterica, no. (%)]	17 (52%)	4 (50%)	>0.999
Pathological fracture at presentation [no. (%)]	7 (21%)	6 (75%)	0.007
Internal fixation [LCP-PHP, no. (%)]	24 (73%)	7 (88%)	0.653
Intraoperative blood loss (ml)	96.67 ± 97.68	242.50 ± 354.67	0.286
Intraoperative transfusion (ml)	43.94 ± 94.17	106.25 ± 161.33	0.324
Number of recurrent cases [no. (%)]	6 (18%)	1 (13%)	>0.999

In our study, bone cysts exhibited a 17% recurrence rate (7/41), with the median time to recurrence being 14.27 months post-surgery (range 9–56 months). Subsequent to allograft bone reimplantation and X3 injection, all individuals achieved bony union at the last assessment. Analysis of potential risk factors indicated no significant correlation between recurrence and parameters such as age, gender, pathology type, presence of preoperative pathological fractures, type of internal fixation, or intraoperative blood loss (*P* > 0.05). Notably, cysts located between the capital femoral epiphysis and the linea intertrochanterica were markedly associated with recurrence (*P* < 0.05) (Table 4).

**Table 4:** Analysis of risk factors for relapse.

	No recurrence (*n* = 34)	Recurrence (*n* = 7)	*P* value
Age at surgery (years)	7.71 ± 2.70	6.27 ± 2.36	0.200
Gender [female, no. (%)]	8 (24%)	2 (29%)	0.556
Staging [active bone cyst, no. (%)]	9 (27%)	4 (57%)	0.181
Location [between the capital femoral epiphysis and the linea intertrochanterica, no. (%)]	6 (18%)	5 (71%)	0.010
Pathology type (ABC), *n* (%)	11 (33%)	2 (29%)	>0.999
Combined pathologic fracture, no. (%)	11 (32%)	2 (29%)	>0.999
Internal fixation [LCP-PHP), no. (%)]	27 (79%)	4 (57%)	0.150
Use of Kirschner pins [no. (%)]	9 (27%)	3 (43%)	0.398
Number of complication cases [no. (%)]	7 (21%)	1 (14%)	0.584
Preoperative RBC (10^12^/L)	4.59 ± 0.48	4.40 ± 0.30	0.331
Preoperative HGB (g/L)	128.32 ± 12.59	122.14 ± 8.40	0.224
Preoperative Hct (%)	37.54 ± 3.70	36.80 ± 2.72	0.620
Operative time (min)	101.21 ± 28.18	111.14 ± 21.27	0.385
Intraoperative blood loss (ml)	125.88 ± 195.91	121.43 ± 96.68	0.954
Intraoperative transfusion (ml)	50.00 ± 103.72	85.71 ± 146.39	0.444
Follow-up time (months)	25.04 ± 19.23	33.64 ± 17.33	0.281

## Discussion

In this study, 41 pediatric proximal femur bone cysts were addressed through lesion curettage, subsequent defect filling with allograft bone and MIIG X3, and, when deemed necessary, internal fixation—yielding commendable clinical and radiographic outcomes. Notably, the cyst's position in the proximal femur was associated with both the onset of pathological fractures and the likelihood of postoperative recurrence. The presence of pathological fractures could prolong surgical time, increase intraoperative hemorrhage, potentially require blood transfusion, and amplify postoperative complication risks. Consequently, proactive surgical treatment is recommended for proximal femur bone cysts.

The proximal femur, subjected to substantial mechanical stress, is particularly vulnerable to the risks of fractures and deformations when compromised by a benign tumor. Lesions in this location present pronounced challenges owing to their distinct anatomical position, and the complexity is amplified when faced with imminent or established fractures (6). Notably, bone cysts often span from the subtrochanteric region to the femoral neck, with the bone cortex typically being attenuated and more prone to fracture. In current practice, an array of fixation methods are employed to bolster the reconstructive strength, facilitating early mobilization in treating pathological fractures or high-risk lesions in the proximal femur. Moreover, it's imperative to account for the unique anatomical and vascular attributes of the femoral neck and head when managing these conditions (6, 9). Guided by our clinical observations, we ardently support surgical interventions that entail complete lesion excision, defect rectification, and firm internal fixation. Such a strategy seeks to foster bone cyst and fracture healing while mitigating potential complications.

Neer et al. (20) documented 24 instances of proximal femoral simple bone cysts (SBCs) concurrent with pathological fractures. These cases underwent treatment via curettage and bone grafting, resulting in a 17% (4 cases) reoperation rate, with re-fracture occurring in 3 out of the 4 reoperated cases. Wilke et al. (21) found that, among 11 patients with proximal femoral bone cysts, 73% (8 patients) initially managed without internal fixation eventually necessitated some iteration of internal stabilization. Similarly, Lin et al. (9) reported a 78% failure rate in initial conservative treatments. Notably, among nine patients who initially abstained from internal fixation, eight necessitated subsequent surgical interventions. Given these findings, an initial internal fixation approach is advocated for children presenting with pathologic fractures in the proximal femur, aiming to curtail the likelihood of follow-up surgeries (9, 21). While this strategy might not expedite the bone cyst healing timeline, it facilitates a swifter return to regular activities and minimizes the logistical and emotional toll of successive surgeries on patients and their kin.

In the series by Wilke et al., 60% (three out of five) of the patients treated with FINs for proximal femoral bone cysts necessitated additional surgeries to switch to longer FINs (21). It is generally observed that the stability of fracture fixation can be significantly improved by placing the end of the lateral intramedullary nail into the greater trochanter and positioning the medial nail as proximally as possible to the epiphysis. It's noteworthy that FINs are infrequently employed for the fixation of femoral neck fractures. Most prior literature has predominantly addressed their application in non-pathological femoral neck fractures, specifically of the Delbet type IV (22, 23). A salient challenge for patients with proximal femoral cysts is the necessity to create a window at the lesion site for its removal, undermining the merits of employing intramedullary nails for minimally invasive interventions. Rigorous curettage stands out as a paramount procedure in the therapeutic regimen for bone cysts (24, 25). Presently, our inclination leans toward using LCP-PHP for internal fixation in cases of fractures or potential fractures instigated by proximal femoral bone cysts. This proclivity stems from the heightened vulnerability of the lesioned area post-fenestration and curettage with a high-speed burr, coupled with the possibility of the lesion compromising the zone buttressed by the distal end of the intramedullary nails.

Schrader et al. (26) conducted a retrospective analysis of 15 pediatric patients presenting with pathological fractures due to benign lesions and observed an average union time of 17 weeks. This stands in contrast to the findings of Erol et al. (7), where the average time to union was reported as 10 weeks, ranging between 8 to 12 weeks. Yet another study pinpointed the mean duration for healing at 4.9 months (9). It is imperative to recognize that the ultimate therapeutic objective extends beyond merely achieving fracture union; it also encompasses the total eradication of the bone cyst.

## Recurrence

Tomaszewski et al. observed a 13.6% recurrence rate in pediatric patients with proximal femoral bone cysts, with each case reappearing within 4–6 months post-surgery (5). In a study by Erol et al., merely 5 of the 58 patients diagnosed with bone cysts exhibited less than 80% obliteration of the cystic defect. Interestingly, four of these patients showed progressive cortical thickening without necessitating reoperation. However, one required subsequent curettage, bone grafting, and additional internal fixation 12 months after the primary procedure (7). Past literature primarily reported recurrence within the initial 2 years, with a minority emerging in the third and fourth years post-intervention (27). At our institution, we noted a 17.1% recurrence rate, with a median recurrence time of 14.27 months (ranging between 9 to 56 months) post-surgery. All these recurrent cases eventually achieved complete healing following extended curettage and bone grafting procedures. Despite our efforts to pinpoint risk factors associated with bone cyst recurrence, we found no direct correlation to either the pathological type or the mode of internal fixation. Yet, it's worth noting that bone cysts located between the capital femoral epiphysis and the linea intertrochanterica exhibited a significantly elevated recurrence rate compared to cysts in other areas.

## Complications

LLD can arise from damage to the proximal femoral epiphysis. Although the distal femoral epiphysis accounts for approximately 66% of the femur's overall longitudinal growth (28), injuries to the proximal segment can impede this growth. Tomaszewski et al. reported LLD in 13.3% (4 out of 30) of patients with proximal femoral bone cysts and tumor-like manifestations, with one undergoing contralateral distal femur epiphysiodesis (5). Schrader et al. documented a 27% prevalence of LLD among 15 pediatric patients afflicted with benign tumor-induced pathological fractures (26). In this study, we identified seven instances of LLD, all ≤20 mm, none of which currently necessitated surgical intervention. Patrikov et al. associated two instances of LLD among six patients with proximal femoral bone cysts and concurrent pathological fractures to potential harm from a 5.0 mm, 130° LCP-PHP traversing the growth plate. They advocated for a 3.5 mm diameter LCP-PHP (8). Contrarily, our findings did not confirm this correlation. Typically, when internal fixation crossed the growth plate, an additional 2 mm K-wire was employed, avoiding plate disorganization. Biannual standing full-leg X-rays are recommended until skeletal maturity.

AVN stems from a series of pathological processes due to disruptions in the blood supply to portions of the femoral head (29). Our findings indicated that using LCP-PHP and elastic intramedullary nailing for internal fixation imparted minimal disruption to the femoral head's vascular supply. Furthermore, no cases of femoral head necrosis emerged in our study.

In the study by Lin et al., the incidence of coxa vara following pathological fractures of the proximal femur was 14% (9). In contrast, a systematic literature review reported an incidence rate of 18.5% after non-pathological fractures (30). These findings suggest that children with pathological fractures of the proximal femur do not face a heightened risk of coxa vara development compared to those with non-pathological fractures. Jamshidi et al. administered treatment to 14 pediatric cases of proximal femoral SBCs using a proximal locking plate combined with a fibular strut allograft. Out of these, one case manifested a mild coxa vara deformity (10). In our study, however, three cases exhibited a coxa vara deformity, accompanied by shortening of the femoral neck. Potential contributing factors to this outcome may include growth plate disorganization, suboptimal fracture reduction, or insufficiently secure stabilization of fracture ends through internal fixation.

Given that children are in a developmental phase with their proximal femoral epiphysis still open, the decision on internal fixation needs to strike a balance between ensuring fracture stability and minimizing potential harm to the growth plate. The distinctive anatomical position of the proximal femur renders treatment of its bone cysts unique, necessitating proactive intervention even if fractures aren't present (21). Utilizing the modified Watson-Jones surgical approach offers a clear view of the lesion site, facilitating fracture reduction under direct visualization. In our institution, children with bone cysts coexisting with pathological fractures experienced notably more intraoperative blood loss and prolonged surgical durations compared to those without such fractures. Moreover, postoperatively, these children exhibited a higher incidence of aberrant proximal femoral morphology. Consequently, we advocate for aggressive management of proximal femoral bone cysts, especially in cases where the cyst index exceeds 3.5, or where the cyst encompasses more than 85% of the long bone's diameter, signifying elevated fracture risks.

## Limitations

This study is influenced by the constraints inherent to retrospective designs. Moreover, given that multiple physicians administered the treatments to these pediatric patients, potential heterogeneity might have been introduced. Our institution's infrequent application of FIN renders comparison of outcomes across various internal fixation methods challenging. While patients were monitored for a minimum of one year, extended follow-up remains imperative to gauge both cyst recurrence and protracted complications.

## Conclusion

In pediatric patients, proximal femoral bone cysts can be effectively treated through lesion curettage, defect filling with allograft bone and MIIG X3, complemented by a selective approach to internal fixation. The presence of pathologic fractures preoperatively can lead to extended surgical durations, elevated intraoperative bleeding, and a heightened likelihood of requiring blood transfusions and experiencing post-operative complications. Therefore, proximal femoral bone cysts that have not yet suffered a pathologic fracture should be closely observed and actively treated.

## Data Availability

The original contributions presented in the study are included in the article/Supplementary Material, further inquiries can be directed to the corresponding authors.
